# Pediatric endurance and limb strengthening for children with cerebral palsy (PEDALS) – a randomized controlled trial protocol for a stationary cycling intervention

**DOI:** 10.1186/1471-2431-7-14

**Published:** 2007-03-21

**Authors:** Eileen G Fowler, Loretta M Knutson, Sharon K DeMuth, Mia Sugi, Kara Siebert, Victoria Simms, Stanley P Azen, Carolee J Winstein

**Affiliations:** 1 Department of Orthopedics, UCLA/Orthopaedic Hospital Center for Cerebral Palsy, Los Angeles, CA, USA; 2 Department of Physical Therapy, Missouri State University, Springfield, MO, USA; 3 Division of Biokinesiology and Physical Therapy, University of Southern California, Los Angeles, CA, USA; 4 Department of Preventive Medicine, Keck School of Medicine, University of Southern California, Los Angeles, CA, USA

## Abstract

**Background:**

In the past, effortful exercises were considered inappropriate for children with spastic cerebral palsy (CP) due to concern that they would escalate abnormalities including spasticity and abnormal movement patterns. Current scientific evidence indicates that these concerns were unfounded and that therapeutic interventions focused on muscle strengthening can lead to improved functional ability. However, few studies have examined the potential benefits of cardiorespiratory fitness exercises in this patient population.

**Methods/design:**

The rationale and design of a randomized controlled trial examining the effects of a stationary cycling intervention for children with CP are outlined here. Sixty children with spastic diplegic CP between the ages of 7 and 18 years and Gross Motor Function Classification System (GMFCS) levels of I, II, or III will be recruited for this study. Participants will be randomly assigned to either an intervention (cycling) or a control (no cycling) group. The cycling intervention will be divided into strengthening and cardiorespiratory endurance exercise phases. During the strengthening phase, the resistance to lower extremity cycling will be progressively increased using a uniquely designed limb-loaded mechanism. The cardiorespiratory endurance phase will focus on increasing the intensity and duration of cycling. Children will be encouraged to exercise within a target heart rate (HR) range (70 – 80% maximum HR). Thirty sessions will take place over a 10–12 week period. All children will be evaluated before (baseline) and after (follow-up) the intervention period. Primary outcome measures are: knee joint extensor and flexor moments, or torque; the Gross Motor Function Measure (GMFM); the 600 Yard Walk-Run test and the Thirty-Second Walk test (30 sec WT).

**Discussion:**

This paper presents the rationale, design and protocol for Pediatric Endurance and Limb Strengthening (PEDALS); a Phase I randomized controlled trial evaluating the efficacy of a stationary cycling intervention for children with spastic diplegic cerebral palsy.

## Background

Cerebral palsy (CP) is caused by an insult to the developing brain. The prevalence is between 1.5 and 2.5 per 1,000 live births in developed countries [1] and spastic diplegia is the most common form [2]. These children exhibit weakness [3-7] and low endurance [8-12]. Historically, programs to promote physical fitness, including strengthening and cardiorespiratory fitness exercise, were discouraged for patients with spastic CP due to the concern that spasticity and abnormal movement patterns would worsen [13]. Scientific evidence has not supported this concern [14-16] and current research indicates that resistive exercise is an effective intervention to improve strength and function in children with CP [16-21]. However, a recent review of strengthening intervention studies concluded that more research of higher quality is needed [20]. Only one of the studies reviewed was a randomized controlled trial (RCT). The magnitude of the effect size for strength changes varied widely across the studies. Outcome variations may be due to methodological differences in intervention intensity, frequency and duration. There is considerably less research examining the effectiveness of cardiorespiratory fitness for children with CP.

Cycling is a rehabilitation tool often used by physical therapists to improve strength and cardiorespiratory fitness [22] and has been promoted as an appropriate exercise to improve fitness for persons with CP [23,24]. Stationary cycling programs can provide progressive resistance exercise for lower extremity musculature. Normative adult data has demonstrated significant muscle recruitment, based on electromyography (EMG), during cycling for the major lower extremity joint extensors and flexors [25]. Mean recruitment was at least 50% of maximum EMG for the soleus, gastrocnemius, hamstring, vastus medialis/lateralis, rectus femoris and gluteus maximus muscles during the propulsive phase (limb extension) and tibialis anterior muscle during the recovery phase (limb flexion) [25]. Kaplan [26] studied pedaling smoothness and EMG in children with and without CP during a single stationary cycling session. Fifteen children with spastic diplegic CP and 15 nondisabled children were assessed. All children required practice to familiarize themselves with the bike and seven of the children with CP required physical guidance to prevent backward motion at the top of the pedaling cycle. Greater ankle muscle coactivation was observed for all pediatric subjects as compared to that reported for nondisabled adults. Prolonged periods of knee and ankle muscle coactivation occurred in children with CP as compared to controls.

There is limited literature supporting cycling as a therapeutic intervention for children with CP. A tricycle designed to increase hip extensor recruitment in children with CP has been described [27,28] and tested with a small number of children. Five boys with CP between the ages of five and seven years participated in an eight-week home program of tricycle riding. Children cycled an average of 30 minutes per day. Four of the five parents gave the tricycle a high ranking indicating that their children exhibited greater mobility. One study found improved cardiorespiratory fitness, based on a measure of oxygen uptake, in a group of 20 children with spastic and dyskinetic CP following an aerobic exercise intervention that included lower limb cycling [29]. Exercise frequency was three times weekly for 20 minutes. The precise cycling mode and the duration of the exercise, 1.6 – 16 months, varied widely but these results demonstrate that improvement in cardiorespiratory fitness is possible in this population.

Stationary cycling interventions for children with CP warrant further examination as they have the potential to improve strength and cardiorespiratory fitness with minimal requirements for balance and motor control. The purpose of this study is to evaluate the effectiveness of a stationary cycling intervention for children with spastic diplegic CP using a RCT design. We hypothesize that those children randomized to the cycling group will demonstrate a significant improvement in muscle strength, walking endurance, gross motor function and health related quality of life compared to those randomized to the control (no-cycling) group.

## Methods/design

The PEDALS Project for children with CP was designed as a Phase I RCT due to the paucity of previous research that has critically examined the effect of stationary cycling for children with CP. PEDALS is one of four projects hosted by the Physical Therapy Clinical Research Network (PTClinResNet), a member of the Inventory and Evaluation of Clinical Research Networks (IECRN)[30] PTClinResNet was established in 2002 to support research that examines the efficacy of physical therapy interventions. This national network supports evidence-based research across disabilities by linking collaborators across the country representing a range of disciplines, with a coordinating center at the University of Southern California. The organizational infrastructure of PTClinResNet includes a central data management and analysis team, a scientific advisory panel, and a data monitoring and safety board. PTClinResNet uses the International Classification of Functioning (ICF) framework to link outcome measures within and across projects [31,32]. The ICF contains three domains of human function: body function and structure, activity and participation. Body function and structure refers to the physiological function of body systems and the anatomical parts of the body. Activity refers to the performance of a task or action by the whole person. Participation refers to an individual's involvement in life situations. Environmental and personal contextual factors are also included in this framework.

The Institutional Review Boards of the University of Southern California, University of California at Los Angeles, Missouri State University, the State of California, Orthopaedic Hospital in Los Angeles, California and CoxHealth Hospital in Springfield, Missouri granted approvals for this research protocol. This study has a pre-post, control group design with single-blinding. Subjects will be randomly assigned to intervention versus control groups. There are two stratifying variables; age (younger, 7–11 and older, 12–18 years) and selective voluntary motor control ability (good versus fair). Randomization to the intervention or control group will be blocked by these two variables. The assessment of selective motor control ability for children with CP has been described by Staudt and Peacock [33]. Good selective motor control is defined as the ability to isolate knee and ankle movement out of synergy (knee extension with the hip in flexion and ankle dorsiflexion with the knee in extension). Fair selective motor control is defined as the ability to isolate knee movement without obligatory hip movement. Only subjects who demonstrate good selective motor control bilaterally will be placed in the good selective motor control category for stratification. Subjects who have poor selective motor control (cannot isolate knee and ankle joint motion out of synergy) bilaterally will be excluded from the study. All others will be placed in the fair selective motor control category.

Interventions will be performed by physical therapists in community clinics throughout Southern California and Southwest Missouri. Evaluations will take place at the University of Southern California or Missouri State University and will be performed by physical therapists blinded to subject group randomization. Evaluation and intervention physical therapists will attend instructional sessions and receive a written manual of procedures specific to either evaluation or intervention protocols. Performance will be videotaped during mock sessions and scored by members of a standards committee using a checklist of critical components for each protocol. To participate in the study, therapists must achieve a minimum score of 90% accuracy. In addition, they will be provided with feedback for aspects of the protocol that are not performed optimally.

### Subjects

Subjects will have a diagnosis of spastic diplegic CP. Inclusion criteria will be: 1) age between 7 and 18 years; 2) ability to follow simple verbal directions; 3) good or fair selective motor control for at least one lower limb and 4) ability to walk independently indoors, with or without assistive devices (Levels I-III of the Gross Motor Function Classification System (GMFCS) [34]. Exclusion criteria will be: 1) orthopedic surgery, neurological surgery or baclofen pump implantation within the preceding 12 months; 2) botulinum toxin injections within the preceding three months; 3) serial casting or new orthotics within the preceding three months; 4) initiating oral medications that affect the neuromuscular system, e.g. baclofen, within the preceding three months; 5) onset of physical therapy, exercise, sport activity or change in assistive devices for walking within the preceding three months; 6) inability or unwillingness to maintain age appropriate behavior; 7) serious medical conditions such as cardiac disease, diabetes or uncontrolled seizures; 8) current participation in a fitness program that includes cardiorespiratory endurance exercise, at least one time per week and 9) significant hip, knee or ankle joint contractures preventing passive movement of the lower limbs through the pedaling cycle.

Subjects will be recruited from southern California and southwest Missouri via flyers and brochures placed in clinics and schools and by postings on disability related web sites. When a potential subject contacts the investigators, a telephone screening will be performed. Subjects meeting the inclusion and exclusion criteria assessed during the telephone screening will receive an in-person screening to determine GMFCS level and selective motor control ability. Additionally, the child will be assessed on the stationary bicycle to ensure that their lower extremity joint range of motion is sufficient to move through the pedaling cycle.

If the subject qualifies during the in-person screening, enrollment procedures will occur. An investigator will explain the informed consent, medical release, photo consent and a summary of the Health Insurance Portability and Accountability Act of 1996 (HIPAA) policy to the participants and their guardians. Testing purposes and procedures, the randomization process, potential risks and benefits for participants and responsibilities of both the participants and the research team will be discussed and questions answered. If the individual and their parent/guardian decide to proceed with enrollment, the consent form will be signed and an enrollment form, containing the subject's age and selective motor control ability, will be submitted to the PTClinResNet Data Management Center for subject randomization. Families will be notified of their child's assignment to the control or intervention group following the baseline evaluation.

### Sample size

Sixty subjects (30 cycling intervention, 30 no cycling control) will be recruited for this study. A sample size of 30 in each group will have 80% power to detect a moderate effect size of 0.7 (as well as a minimal clinically important difference) using a two group t-test with a 0.05 two-sided significance level. Power analyses were performed using joint moment data outcomes following an isokinetic strengthening intervention for children with CP [16]. A 10% subject attrition rate was factored into the analyses.

### Stationary bicycle

**Figure 1:**
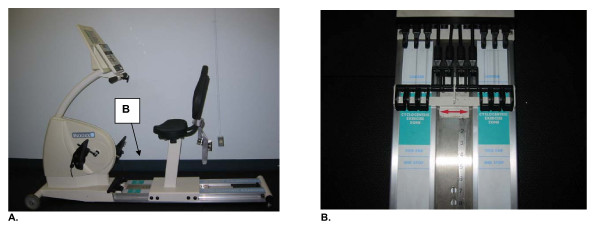
**A**. The Biodex Cyclocentric Semi-Recumbent Cycle has a wide-based seat with an optional seat belt. The seat is released by a handle located below the front panel allowing it to slide back along the track when the subject pushes forward on the pedal. **B**. Ten tensioning cords (10 lbs per cord) located at the base of the stationary bicycle provided progressive levels of loading. In this photograph, four of the cords are engaged for a total load of 40 lbs resistance. The arrows point to a location within the "cyclocentric exercise zone" when the seat is positioned for optimal lower limb range of motion during resisted cycling.

The stationary bicycle in this study was designed for rehabilitation and allows the subject to begin cycling with minimal resistance (Biodex Lower Body Cycle, Biodex Medical Systems, Inc, Shirley, New York) (Fig. 1). This bike uses a semi-recumbent design with a wide padded seat and seat belt, back support, and foot straps attached to the pedals. There are two mechanisms to provide resistance to the lower extremities during cycling: 1) standard cycling and 2) cyclocentric cycling. During standard cycling, the bicycle seat is locked in place. The exercise mode is adjustable from a front operation panel using two different options: "Aerobic exercise – constant power" or "Strengthening exercise – isokinetic". The aerobic exercise mode employs an effort level control from 1–30. The strength exercise mode uses a speed control increment from 25 to 120 degrees per minute.

Cyclocentric cycling is a unique limb-loaded feature of this stationary bicycle and its use for rehabilitation following stroke has been described by Brown and colleagues [35]. To use this feature, the seat is released and allowed to slide forward and backward along a linear track. The seat is released by pulling a handle located just below the bicycle's front panel. Up to ten tensioning cords (10 lbs of force per cord) can be attached to the base of the seat acting to pull the seat forward, thereby passively flexing the subject's lower extremities. Extension of one lower limb is necessary to resist the excessive flexion caused by the tensioning cords, and to maintain the seat within a desired range called the "cyclocentric exercise zone". This optimal forward-backward position for the seat is indicated by arrows pointing to a location within a green colored zone along the linear track located at the base of the bicycle (see Figure 1a and 1b). This zone is visible to the subject during cycling.

### Cycling intervention protocol

Thirty exercise sessions will occur over a 12-week period. The optimal frequency is three times per week over a ten-week period; however, this alternative schedule provides flexibility for vacations, illnesses or other events. Each session will last approximately 60 minutes. Subjects will be asked to wear shorts and tennis shoes during each session for comfort, safety and assessment purposes. They will receive individualized instruction for an independent self-stretching exercise program for bilateral hip flexor, knee extensor, knee flexor, and ankle plantar flexor muscles as a 5–10 minute warm-up prior to cycling. Additional muscle groups that exhibit reduced range of motion may be included in the stretching program for individual subjects. The cycling intervention will be divided into two phases: 1) Lower extremity strengthening and 2) Cardiorespiratory endurance.

#### Phase 1: lower extremity strengthening

The seat location will be adjusted to ensure a knee joint angle of between 15 and 20 degrees of flexion when the knee is maximally extended during cycling. Subjects will be instructed to hold onto the side of the seat because it is possible to slide the seat backwards by pushing with the upper extremities when using the alternate hand rest position on the front of the bicycle. Lower extremity resistance training will begin with the attachment of one tensioning cord. The seat will be released and the subject instructed to begin cycling while keeping the seat within the desired cyclocentric zone. If the subject has difficulty transferring resistance from one limb to the other, the therapist may assist by stabilizing the seat in the desired cyclocentric zone as the lower limb moves from maximum flexion into extension at the top of the pedal revolution. Difficulty during this transition results in a "jerking forward" of the seat rather than a smooth rhythmic cycling motion. If the subject continues to have difficulty with this transition, the therapist may physically assist the foot in moving forward through the top of the pedaling cycle. The subject will be instructed to avoid "locking" the knees in full extension near the bottom of the pedaling cycle, a strategy that might occur as a compensation for weakness. If this deviation persists, a physical block will be slid along the track and positioned behind the seat to prevent excessive knee extension. Once the subject is able to cycle in a smooth pattern without difficulty for ten complete pedaling revolutions, a second tensioning cord will be engaged increasing the resistance to 20 lbs. The above protocol will be repeated until the subject either cannot cycle at the next higher cord increment or the cycling pattern is not smooth. This level of resistance will be recorded as the maximum cord level for the session.

During subsequent sessions, the subject will begin with a minimum of 20 revolutions at cord levels below the previous session's maximum. They will progress to the maximum cord level, gradually increasing the number of revolutions. During each session, the minimum and maximum number of tensioning cords and the corresponding number of revolutions will be recorded. The maximum resistance possible is 10 cords, or 100 lbs of force. The total duration of this phase of the intervention is approximately 10–15 minutes.

#### Phase 2: cardiorespiratory endurance training

During the first intervention session, the subject will be instructed in the use of the Children's Effort Rating Table (CERT) [36]. The CERT uses a 1 to 10 numeric scale, with 1 corresponding to "very, very easy," to 10 corresponding to "so hard I'm going to stop." At the beginning of each intervention session, resting heart rate (HR) will be recorded after a period of quiet sitting. A target HR range of 70 to 80% maximum HR will be calculated for each session using the Karvonen Formula [37] (Target HR = [(HRmax - HRrest) × (0.70 or 0.80)] + HRrest; where HRmax = 220 - age). A sensor placed on the ear or the chest will monitor the subject's HR. The therapist will assess the radial pulse to confirm accuracy of the electronic HR monitor prior to initiating cycling. If the electronic HR monitor readout is different, adjustments of sensor placement will be made until agreement is found.

To begin the cardiorespiratory endurance phase, the limb-loading feature will be disengaged and the bike locked in a stable position. The seat will be positioned to ensure that the subject's knee joint is in 15 – 20 degrees of flexion when maximally extended. Cycling resistance will be adjusted using the "Aerobic exercise – constant power" mode. Cycling will begin at a low level and be adjusted up or down according to the subject's ability. If the subject is able to cycle for 10 consecutive minutes within the target HR range, the therapist will switch to the "Strength exercise – isokinetic" mode with a setting of 60 cycles per minute for a two-minute period, thus increasing the intensity of the resistance. The goal for the initial session is 15 minutes of cycling. A variety of motivational strategies will be used during the intervention to promote continued cycling and to increase intensity of effort. Therapists will place the HR monitor in front of the subject and encourage them to cycle faster in order to increase their HR. Additional strategies include verbal encouragement, listening to music, pretend play and counting the number of lower extremity revolutions possible while maintaining a given HR level or exercise level setting on the bicycle. The child will be asked to describe their perceived exertion throughout the cycling session using the CERT. If the HR is below target range but the subject is cycling at a high rate, the constant level resistance will be increased. If a subject expresses a high level of fatigue, or a CERT level of 9 or 10, the constant level resistance will be decreased or the subject will be instructed to slow the pedaling rate. Subjects will be encouraged to gradually increase their exercise duration to a maximum of 30 minutes over the thirty sessions. A cool-down period will occur at the end of the intervention as the subject pedals without resistance until his or her HR decreases to within 20 beats above baseline. Therapists will be provided with a worksheet to record HR and CERT levels. For each session, an intervention form will be completed containing the following information: 1) baseline HR; 2) target HR range; 3) aerobic exercise level setting; 4) the subject's typical exercise HR (TEHR); 5) duration of the TEHR; 6) time to reach TEHR; 7) total cycling duration and 8) CERT level while at TEHR. If a range of values are observed or reported for these parameters, the most representative for the session will be recorded.

### Outcome measures

#### Outcome data collection sessions

For the Los Angeles site, data collection will take place at the Division of Biokinesiology and Physical Therapy at the University of Southern California and the Francisco Bravo Medical Magnet High School Gymnasium, Los Angeles Unified School District. Missouri site data collection will take place at Missouri State University. An interpreter will be present for parents or guardians who are non-English speaking. Measurements of subject height and weight will be obtained during each session. For the control group, follow-up evaluations will be scheduled within 10–12 weeks after the baseline evaluation. For the intervention group baseline data will be collected within a one-month period prior to the initiation of intervention sessions. Follow-up data will be collected within a two-week period following the last intervention session.

#### Outcome data measurements

Outcome measures for this study include assessments at the body function and structure, activity and participation levels of the ICF. Primary outcome measures are: 1) knee joint extensor and flexor torque; 2) the Gross Motor Function Measure (GMFM) Sections D: Standing and E: Walking, Running and Jumping [38]; 3) the 600 Yard Walk-Run test [39] and 4) the Thirty-Second Walk test (30 sec WT) [40]. Secondary measures are: 1) the Pediatric Outcomes Data Collection Instrument (PODCI) [41], 2) the Pediatric Quality of Life Inventory™ (PedsQL) [42-44] and 3) instrumented gait analysis.

Knee joint extensor and flexor moments, commonly referred to as joint torque in a clinical setting, are measures at the body function and structure level of the ICF. A Kin-Com dynamometer (Chattanooga Group Inc., Hixson, TN) will be used for data collection at the Los Angeles site and a Biodex Multijoint System (Biodex Medical Systems Inc., Shirley, NY) at the Missouri site. The subject will be seated with the trunk slightly reclined. Straps at the waist, chest, thigh and distal aspect of lower leg will be used to secure the subject to the testing device. The axis of the device's moving arm will be aligned with the subject's knee joint center. The subject will be directed to observe the computer screen for visual feedback while being verbally encouraged by the investigator to provide maximum effort. Five repetitions of knee joint extension and flexion at 0, 30, 60 and 120 degrees/second will be performed bilaterally. The peak joint moment for each group of five contractions will be determined.

The GMFM assesses performance at the activity level of the ICF. It has been shown to be valid [45] and reliable [46] for assessing children with CP. Two of the five dimensions, Dimension D: Standing (13 items) and Dimension E: Walking, running, and jumping (24 items) will be tested. GMFM sessions will be videotaped to permit review should a question about a particular score arise. Subjects will wear shorts and be tested barefoot and without assistive devices or ankle-foot orthoses. The evaluation therapists will follow the procedures outlined in the Gross Motor Function Measure User's Manual [38] for testing and scoring. Each item will be scored as 0 = does not initiate, 1 = initiates, 2 = partially completes, 3 = completes or NT = not tested. The data will be analyzed using the GMFM-66 Ability Estimator Software. The advantage of the software is the conversion of the ordinal data into an interval scale. This will allow for a more accurate estimate of the child's ability and provide a measure that is equally responsive to change across the spectrum of ability levels [38].

The 600 yard walk-run test [39] assesses walking and/or running endurance at the activity level of the ICF. This test is an indirect measure of cardiorespiratory fitness and is reflective of a child's ability to participate in play and sport activities. It is a standardized physical fitness test developed for school-age children. Fernhall et al. [39] used this test for children with intellectual disabilities and found a high correlation with laboratory measures of peak V0_2_. During this test, children are asked to complete a 600 yard distance as fast as possible by running, walking or a combination of both. The distance required will be clearly explained so they may pace themselves. Orange cones will be placed to visibly mark the perimeter of the circular path. Subjects will be encouraged verbally to continue walking or running until they complete the 600 yard distance. If a subject cannot complete this distance within 15 minutes or stops for more than five seconds, the test will be stopped. At the end of the test, the distance completed and the time will be recorded. Distances of less than 600 yards will be measured using a distance-measuring wheel. Outcomes include the distance the child is able to complete, the time for completion and the speed.

The 30 sec WT assesses walking function at the activity level of the ICF and is reflective of a child's ability to walk within the school environment. Normative data for 227 children between the ages of six and 13 years are available for comparison [40]. This test will be administered in a gymnasium by asking the subject to walk at a comfortable speed and to stop when 30 seconds have elapsed. Children will be instructed to walk as if they were the leader in a line at school. The examiner will monitor time using a stopwatch. When 30 seconds have elapsed, the examiner will instruct the subject to "freeze" and not move until his or her foot position is marked. The distance from the starting line to the heel of the forward-most foot will be measured using a distance-measuring wheel. Outcomes will be the total distance walked and walking speed.

The health-related quality of life questionnaires to be used for this study assess multiple domains of function within the ICF but focus on environmental and personal contextual factors. Questionnaires will be administered in an interview format. The PODCI Scale was developed to assess health-related quality of life in children from 7 – 19 years of age. It demonstrated good reliability, construct validity and sensitivity to change over a 9-month period [41]. This questionnaire will be completed by the parent, or guardian, and all adolescents (11–18 years). Individuals will be asked to respond to questions by choosing from a list of possible answers. A Spanish language version of this tool will be available. Interpreters will be made available for other languages. There are eight scales: upper extremity and physical function, transfer and basic mobility, sports/physical functioning, pain/comfort, treatment expectations, happiness, satisfaction with symptoms and a global functioning scale (a combined scale calculated from the first four scales). All components are transformed to a 0–100 scale for analyses.

The PedsQL is a health related quality of life instrument specifically designed for children with disability [42-44]. It has been found to be a reliable and valid tool for children aged two to 18 years and was found to be sensitive to age, acute versus chronic disability and unaffected children versus those with disability [42]. This questionnaire will be administered to all subjects in the study. Children will be interviewed using a self-report questionnaire separately from their parents to avoid parental influence. Three different age versions will be used: Young children (5–7 years), Children (8–12 years) and Teen (13–18 years). The children will be asked "In the past one month, how much of a problem has this been for you ...?". A three point Likert scale is used for the Young Child category with responses ranging from 0 (Never) to 4 (Almost Always) and a five point scale is used for the other two age categories. Four separate dimensions are assessed: Physical functioning, Emotional functioning, Social functioning and School functioning. The latter three dimensions are combined to create a Psychosocial Health Summary Score. Items are reverse scored and linearly transformed to a 0–100 scale for analyses.

Gait data will be collected in the Musculoskeletal Biomechanics Research Laboratory at the University of Southern California for a subset of children. Subjects will wear shorts and be barefoot during walking trials at self-selected and maximum walking speed. Hand-held assistance will be allowed, as needed, for safety. Three-dimensional kinematics will be acquired using an 8-camera VICON motion analysis system (Oxford, UK) with a sampling frequency of 60 Hz. Ground reaction forces will be acquired with 3 AMTI force platforms (Watertown, MA) imbedded in the walkway. Joint angles and net joint moments (inverse dynamics approach) will be computed using Visual 3D software (Gaithersburg, MD). Hip, knee and ankle kinematics and temporal data will be calculated for all trials. Joint kinetics will be calculated for trials where the subject's foot makes isolated contact with the force plate.

### Physical activity calendars

All subjects will be provided with calendars and stickers at their initial baseline evaluation to chart their daily physical activity levels throughout the 12-week intervention period. These data are a measure at the ICF level of participation and will inform the investigators of periods of illness or injury that could affect data interpretation. Activities will be classified into four different levels: high, moderate, low and bedrest. The subject will be instructed to place a sticker on each day of the calendar that corresponds to their participation in the following activity levels. A high activity level is defined as participating in activities such as running/jogging, contact sports, hiking, dancing, climbing stairs or biking for over a one-hour period. A moderate activity level is defined as participating in the above activities over a 30-minute period or activities such as swimming, skateboarding, scooter riding or walking for approximately one-hour. A low activity level indicates a sedentary day with activities such as schoolwork, television or computer games. Bedrest indicates that a child is inactive for the entire day due to illness or injury. A folder will be given to the family with calendars, written instructions, colored stickers and contact information for the investigators. Materials are available in English and Spanish. Subjects randomized to the cycling intervention group will be instructed to exclude the cycling intervention when recording their daily activity levels. They will begin calendar recording following their baseline data collection and will return them at the follow-up evaluation session.

### Data analyses

Student t-tests (if normally distributed) or Wilcoxon rank sum tests (if non-normal) will be used to contrast the change from baseline within and between the intervention and control groups for the primary hypotheses. Analysis of covariance procedures will be used to contrast changes adjusting for selective voluntary motor control (good versus fair) and age group (younger, 7–11 years) and (older, 12 – 18 years) for primary outcome measures.

### Incentive for participation

All children will receive an adapted tricycle, bicycle, or stationary bicycle once they have completed their participation in the study. This will provide all subjects with the opportunity to improve their physical health through cycling and help minimize subject attrition.

## Discussion

We have presented the design and rationale for a Phase I RCT assessing the effects of a cycling intervention on joint torque generating capacity, gross motor functional performance, cardiorespiratory fitness and health related quality-of-life outcome measures in children with spastic diplegic cerebral palsy. The results of this trial will be presented as soon as they become available.

## Abbreviations

PEDALS: Pediatric endurance and limb strengthening for children with cerebral palsy

CP: cerebral palsy

GMFCS: Gross motor function classification system

GMFM: Gross motor function measure

30secWT: Thirty-Second Walk test

RCT: randomized controlled trial

EMG: electromyography

PTClinResNet: Physical therapy clinical research network

IECRN: Inventory and evaluation of clinical research networks

ICF: International classification of functioning

HIPAA: Health insurance portability and accountability act of 1996

CERT: Children's effort rating table

HR: heart rate

TEHR: typical exercise heart rate

PODCI: Pediatric outcomes data collection instrument

PedsQL: Pediatric quality of life inventory™

## Competing interests

The author(s) declare that they have no competing interests.

## Authors' contributions

EF, LK and SD were responsible for the design of this study. EF took primary responsibility for the preparation of this manuscript. VS, KS and MS serve as research coordinators, blinded evaluators and contributed to aspects of the protocol design. SA provided statistical consultation. CW contributed to the design of the Network, PTClinResNet. SA and CW had primary responsibility for the design of the data management center. All authors read and approved the final manuscript.

## Pre-publication history

The pre-publication history for this paper can be accessed here:



## References

[B1] Paneth N, Hong T, Korzeniewski S (2006). The descriptive epidemiology of cerebral palsy. Clin Perinatol.

[B2] Bax M, Tydeman C, Flodmark O (2006). Clinical and MRI correlates of cerebral palsy: the European Cerebral Palsy Study. Jama.

[B3] Brown JK, Rodda J, Walsh EG, Wright GW (1991). Neurophysiology of lower-limb function in hemiplegic children. Dev Med Child Neurol.

[B4] Elder GC, Kirk J, Stewart G, Cook K, Weir D, Marshall A, Leahey L (2003). Contributing factors to muscle weakness in children with cerebral palsy. Dev Med Child Neurol.

[B5] Engsberg JR, Ross SA, Olree KS, Park TS (2000). Ankle spasticity and strength in children with spastic diplegic cerebral palsy. Dev Med Child Neurol.

[B6] Rose J, McGill KC (2005). Neuromuscular activation and motor-unit firing characteristics in cerebral palsy. Dev Med Child Neurol.

[B7] Wiley ME, Damiano DL (1998). Lower-extremity strength profiles in spastic cerebral palsy. Dev Med Child Neurol.

[B8] Campbell J, Ball J (1978). Energetics of walking in cerebral palsy. Orthop Clin North Am.

[B9] Hoofwijk M, Unnithan V, Bar-Or O (1995). Maximal treadmill performance in children with cerebral palsy. Pediatric Exercise Science.

[B10] Morgan D, Keefer DJ, Tseh W, Caputo JL, Craig I, Griffith G, Vint P (2005). Walking energy use in children with spastic hemiplegia. Pediatric Exercise Science.

[B11] Rose J, Gamble JG, Medeiros J, Burgos A, Haskell WL (1989). Energy cost of walking in normal children and in those with cerebral palsy: comparison of heart rate and oxygen uptake. J Pediatr Orthop.

[B12] Unnithan VB, Dowling JJ, Frost G, Bar-Or O (1996). Role of cocontraction in the O2 cost of walking in children with cerebral palsy. Med Sci Sports Exerc.

[B13] Bobath K (1980). A Neurophysiological Basis for the Treatment of Cerebral Palsy.

[B14] Dodd KJ, Taylor NF, Graham HK (2003). A randomized clinical trial of strength training in young people with cerebral palsy. Dev Med Child Neurol.

[B15] Fowler EG, Ho TW, Nwigwe AI, Dorey FJ (2001). The effect of quadriceps femoris muscle strengthening exercises on spasticity in children with cerebral palsy. Phys Ther.

[B16] MacPhail HE, Kramer JF (1995). Effect of isokinetic strength-training on functional ability and walking efficiency in adolescents with cerebral palsy. Dev Med Child Neurol.

[B17] Damiano DL, Vaughan CL, Abel MF (1995). Muscle response to heavy resistance exercise in children with spastic cerebral palsy. Dev Med Child Neurol.

[B18] Damiano DL, Kelly LE, Vaughn CL (1995). Effects of quadriceps femoris muscle strengthening on crouch gait in children with spastic diplegia. Phys Ther.

[B19] Darrah J, Wessel J, Nearingburg P, O'Connor M (1999). Evaluation of a community fitness program for adolescents with cerebral palsy. Pediatr Phys Ther.

[B20] Dodd KJ, Taylor NF, Damiano DL (2002). A systematic review of the effectiveness of strength-training programs for people with cerebral palsy. Arch Phys Med Rehabil.

[B21] McCubbin JA, Shasby GB (1985). Effects of isokinetic exercise on adolescents with cerebral palsy. Adapted Physical Activity Quarterly.

[B22] Gregor R, Fowler EG, Zachazewski J, Magee D and Quillen W (1996). Biomechanics of Cycling. Athletic Injuries and Rehabilitation.

[B23] United Cerebral Palsy Research and Education Foundation. Exercise Principles and Guidelines for Persons with Cerebral Palsy and Neuromuscular Disorders 1999..

[B24] Rimmer JH (2001). Physical fitness levels of persons with cerebral palsy. Dev Med Child Neurol.

[B25] Ryan MM, Gregor RJ (1992). EMG profiles of lower extremity muscles during cycling at constant workload and cadence. J Electromyography Kinesiol.

[B26] Kaplan SL (1995). Cycling patterns in children with cerebral palsy. Dev Med Child Neurol.

[B27] King EM, Gooch JL, Howell GH, Peters ML, Bloswick DS, Brown DR (1993). Evaluation of the hip-extensor tricycle in improving gait in children with cerebral palsy. Dev Med Child Neurol.

[B28] Bloswick DS, Brown D, King EM, Howell G, Gooch JR (1996). Testing and evaluation of a hip extensor tricycle for children with cerebral palsy. Disabil Rehabil.

[B29] Berg K (1970). Effect of physical training of school children with cerebral palsy. Acta Paediatr Scand Suppl.

[B30] Inventory and Evaluation of Clinical Research Networks (IECRN) https://www.clinicalresearchnetworks.org/.

[B31] Jette AM (2006). Toward a common language for function, disability, and health. Phys Ther.

[B32] World Health Organization, International classification of functioning, disability and health, Report by the Secretariat, Fifty-fourth World Health Assembly, Provisional agenda item 13.9, April 9, 2001..

[B33] Staudt LA, Peacock WJ (1995). Selective posterior rhizotomy for treatment of spastic cerebral palsy. Pediatr Phys Ther.

[B34] Palisano R, Rosenbaum P, Walter S, Russell D, Wood E, Galuppi B (1997). Development and reliability of a system to classify gross motor function in children with cerebral palsy. Dev Med Child Neurol.

[B35] Brown DA, Nagpal S, Chi S (2005). Limb-loaded cycling program for locomotor intervention following stroke. Phys Ther.

[B36] Williams JG, Eston R, Furlong B (1994). CERT: a perceived exertion scale for young children. Percept Mot Skills.

[B37] Karvonen MJ, Kentala E, Mustala O (1957). The effects of training on heart rate; a longitudinal study. Ann Med Exp Biol Fenn.

[B38] Russell DJ, Rosenbaum PL, Avery LM, Lane M (2002). Gross Motor Function Measure (GMFM-66 & GMFM-88) User's Manual..

[B39] Fernhall B, Pitetti KH, Vukovich MD, Stubbs N, Hensen T, Winnick JP, Short FX (1998). Validation of cardiovascular fitness field tests in children with mental retardation. Am J Ment Retard.

[B40] Knutson LM, Schimmel PA, Ruff A (1999). Standard task measurement for mobility: thirty-second walk test. Pediatr Phys Ther.

[B41] Daltroy LH, Liang MH, Fossel AH, Goldberg MJ (1998). The POSNA pediatric musculoskeletal functional health questionnaire: report on reliability, validity, and sensitivity to change. Pediatric Outcomes Instrument Development Group. Pediatric Orthopaedic Society of North America. J Pediatr Orthop.

[B42] Varni JW, Seid M, Kurtin PS (2001). PedsQL 4.0: reliability and validity of the Pediatric Quality of Life Inventory version 4.0 generic core scales in healthy and patient populations. Med Care.

[B43] Varni JW, Burwinkle TM, Sherman SA, Hanna K, Berrin SJ, Malcarne VL, Chambers HG (2005). Health-related quality of life of children and adolescents with cerebral palsy: hearing the voices of the children. Dev Med Child Neurol.

[B44] Varni JW, Burwinkle TM, Berrin SJ, Sherman SA, Artavia K, Malcarne VL, Chambers HG (2006). The PedsQL in pediatric cerebral palsy: reliability, validity, and sensitivity of the Generic Core Scales and Cerebral Palsy Module. Dev Med Child Neurol.

[B45] Bjornson KF, Graubert C, Buford V, McLaughlin JF (1998). Validity of the Gross Motor Function Measure. Pediatr Phys Ther.

[B46] Bjornson KF, Graubert CS, McLaughlin JF, Kerfeld CI, Clark EM (1998). Test-retest reliability of the gross motor function measure in children with cerebral palsy. Phys and Occup Ther Pediatr.

